# Transcriptome analysis of the adenoma–carcinoma sequences identifies novel biomarkers associated with development of canine colorectal cancer

**DOI:** 10.3389/fvets.2023.1192525

**Published:** 2023-11-29

**Authors:** Zixiang Lin, Jiatong Zhang, Qi Chen, Xiaohu Zhang, Di Zhang, Jiahao Lin, Degui Lin

**Affiliations:** ^1^National Key Laboratory of Veterinary Public Health Security, College of Veterinary Medicine, China Agricultural University, Beijing, China; ^2^Animal Science and Technology College, Beijing University of Agriculture, Beijing, China

**Keywords:** adenoma–carcinoma sequences, canine colorectal cancer, dynamic expression model, PFKFB3, GTPBP4

## Abstract

The concept of adenoma-to-cancer transformation in human colorectal cancer (CRC) is widely accepted. However, the relationship between transcriptome features and adenoma to carcinoma transformation in canines is not clear. We collected transcriptome data from 8 normal colon tissues, 4 adenoma tissues, and 15 cancer tissues. Differential analysis was unable to determine the dynamic changes of genes but revealed that PFKFB3 may play a key role in this process. Enrichment analysis explained metabolic dysregulation, immunosuppression, and typical cancer pathways in canine colorectal tumors. MFuzz generated specific dynamic expression patterns of five differentially expressed genes (DEGs). Weighted correlation network analysis showed that DEGs in cluster 3 were associated with malignant tissues, revealing the key role of inflammatory and immune pathways in canine CRC, and the S100A protein family was also found to be involved in the malignant transformation of canine colorectal tumors. By comparing strategies between humans and dogs, we found five novel markers that may be drivers of CRC. Among them, GTBP4 showed excellent diagnostic and prognostic ability. This study was the first systematic exploration of transformation in canine CRC, complemented the molecular characteristics of the development and progression of canine CRC, and provided new potential biomarkers and comparative oncologic evidence for biomarker studies in human colorectal cancer.

## 1 Introduction

The third leading cause of cancer-related deaths worldwide is colorectal cancer (CRC) (1). In human patients, most cases of colorectal cancer were sporadic, originating from polyps inside abnormal recesses (2, 3), and canine CRC showed a similar pattern (4–6). Tumorigenesis began with the mutation of intestinal epithelial stem cells in the colon or rectal mucosa, then adenoma, and finally colorectal cancer. Over a period of more than 10 to 15 years, tubular adenoma transformed into adenocarcinoma, and alterations in the Wnt, RAS, TP53, and PI3K-AKT pathways all occurred sequentially (7). The effects of Wnt, P53, and TGF-β signaling pathways have been identified in canine CRC studies (8–11). The development of multi-hit gene models of carcinogenesis, together with a deeper understanding of chromosomal instability, microsatellite instability, and the CpG island methylator phenotype of CRC (12, 13), will help clarify the various genetic and epigenetic alterations underlying the adenoma–carcinoma sequence (14). In canine CRC, Jack Russell Terriers were found to have the same germline APC genetic mutation as humans, and this breed was characterized by a high incidence of hereditary colorectal cancer (15, 16). All studies at this stage showed a high degree of similarity between CRC in dogs and humans. And canine CRC may reveal new molecular mechanisms in human (11, 17). Canine colorectal cancer has similar copy number abnormalities to human colorectal cancer, revealing a strong degree of genetic homology between sporadic canine and human CRCs (18). Similar to human colorectal cancer, overactivation of the WNT signaling pathway is also present in canine colorectal cancer, accompanied by repeated mutations in genes associated with CTNNB1 and TGF-β signaling pathways (17). APC was the most significantly mutated gene in both canine adenomas and adenocarcinomas (Frequent Alteration of the Tumor Suppressor Gene APC in Sporadic Canine Colorectal Tumors). The emergence and development of high-throughput omics technologies have facilitated systematic studies comparing normal and tumor tissues at the gene, mRNA, and protein levels. Four consensus molecular sequences (CMS) of CRC-CMS1 (immunotrait, microsatellite instability), CMS2 (canonical trait), CMS3 (metabolic trait), and CMS4 (mesenchymal trait) – and transitional combinations of traits classification into distinct subtypes – established using gene expression data from 4,151 tumor samples (19). The transcriptome analysis of canine CRC also elucidated the molecular signatures specific to proliferative and aggressive canine tumors, revealing that CMS4 human colon cancer consisted of two subtypes, EMT and crypt-like invasion, with differences in TGF-β signaling pathways and microbial content (17).

Although a variety of differentially expressed genes (DEGs) have been found in CRC, the specific expression patterns of these genes in the pathogenesis of colorectal cancer remain unclear. Furthermore, the mechanisms by which CRC arose and evolved are not fully understood. So, it is essential to identify gene expression patterns associated with CRC development and progression.

In this study, transcriptome analysis of normal colon, adenoma, and colorectal cancer identified changes in signaling pathways during canine colorectal tumor formation, and dynamic analysis revealed five specific gene dynamic expression patterns. These findings correlated with three known markers of cancer: dysregulation of cell metabolism, avoidance of immune disruption, and activation of cancer-related pathways. WGCNA analysis identified the gene modules significantly related to the malignant phenotype of the tumor. It was found that the genes in the malignant module were mainly upregulated, and these genes mainly affected the tissue inflammation and immune process, thus revealing the important role of immunosuppression in the malignant transformation of canine CRC. By using a human-canine comparison strategy, we compared genes with a persistent upregulation pattern in human CRC. Five core genes were identified through analysis, and GTPBP4 was further screened as an adverse prognostic marker for colon cancer with optimal diagnostic ability. None of these genes have been studied much in human tumors and neither in canine tumors. Our analysis and comparative strategies identified these novel potential markers that should be continuously monitored and explored during tumor formation.

## 2 Materials and methods

### 2.1 Transcriptome data acquisition and differential analysis

We downloaded transcriptomic data (number PRJNA418842, PRJNA396033) from the SRA database for canine colorectal tumors and normal canine colon tissue. To obtain high-quality clean reads, fastp (v0.20.0) software was used to trim the adaptor and remove low-quality reads. We used STAR (v2.7.9a) software to compare high-quality clean reads to the Canis_lupus_familiaris reference genome (CanFam3.1). Raw read counts of mRNA genes were obtained as mRNA expression values using featurecount (v2.0.2). Normalization was performed using DESeq2 (v1.30.1), retaining only uniquely mapped reads for HTseq counting. The ComBat-seq method of R Package sva was used to remove the batch effect of RNAseq (20).

DEG was identified using the R package DESeq2 with a false discovery rate (FDR) <0.05 and an absolute log2|fold change| > 1. The GTF annotation database (Ensembl v104) was used for mRNA annotation. Gene Ontology and KEGG Pathway enrichment analysis of differentially expressed mRNAs was performed using the clusterProfiler R package (v3.18.1).

### 2.2 Gene set enrichment analysis

Gene Set Enrichment Analysis (GESA) was performed using GSEA software version 3.0^1^ with the Molecular Signature Database gene set version 6.2. All gene expression data were phenotypically arranged with a number of 1,000. Use the FDR value threshold (*p* < 0.05) to identify pathways corresponding to genes enriched at the top or bottom of the gene set and sort according to the normalized enrichment scores.

### 2.3 Dynamic expression model analysis

The core algorithm of the “Mfuzz” R package is based on Fuzzy C-Means Clustering (FCM), which is used to analyze the time trend of gene expression in the transcriptome data with time series characteristics, and cluster the genes with similar expression patterns. To help understand the dynamic expression patterns of these biological molecules and their connection to function. According to the instruction of R package Mfuzz, soft cluster analysis is performed on transcriptome data (21). C-means clustering was performed using R-pack Mfuzz to map reads per million (FPKM) fragments of LRT-identified deg. to assess dynamic changes in expression pattern.

### 2.4 Establishment of co-expression networks and identification of pathological phenotype-related hub modules using weighted gene co-expression network analysis

Co-expression analysis was performed using the Weighted Gene Correlation Network Analysis (WGCNA) R package, guided by published tutorials. Genes with FPKM < 1 of 22 samples were screened and samples (sample type and the number of samples) were clustered hierarchically based on Euclidean distances calculated from gene expression data and combined with (patient clinical information and trait information). Network topology analysis ensured a scale-free topological network and defined a soft-threshold power of (8). A total of (number of modules without counting gray modules) modules were identified based on a dynamic tree-cutting algorithm with a minModuleSize parameter of 30, and mergeCutHeight parameter of 0.25. For each module, the signature gene (the first component expression of the gene in the module) was identified, and then the correlation between the signature gene and clinical phenotype-related description, and clinical features such as tumor staging and grading were calculated. Genes with high connectivity in each module were considered pivotal genes. The co-expression relationships of each module were analyzed and visualized by Metascape (v3.5.20230101).

### 2.5 Analysis of immune cell infiltration in CRC

In this study, we applied the CIBERSORT algorithm to determine the immune cell subsets of canine tissues. Since the CIBERSORT algorithm is able to analyze nonspecific data and noise, it is superior to conventional deconvolution approaches for evaluating infiltrating immunity and for determining the abundances of specialized cells in the mixed matrix.

### 2.6 Cross-validation with human data sets

Due to the lack of veterinary databases containing information about colorectal tumors in dogs, we selected the proteins most relevant to human CRC and validated them using human samples from the Bioinformatics database. Transcriptome data for human colorectal cancer were obtained from the GEO database (GSE164541). Transcriptome data for ROC analysis and differential expression verification were derived from the TCGA database.^2^

### 2.7 Receiver operating characteristic

The pROC package was used for receiver operating characteristic (ROC) analysis of the data, and the results were visualized with ggplot2. The Area Under Curve (AUC) is often used to evaluate diagnostic tests, and the value range of AUC is generally between 0.5 and 1. The closer the AUC is to 1, the better the diagnostic effect of this variable in predicting the outcome.

### 2.8 Survival analysis

The Kaplan–Meier curve is a graphical method used to present the results of survival analyses and is commonly used to analyze the relationship between survival time and event incidence in patients. The optimal cutoffs for classifying patients into high and low gene expression groups were automatically calculated and survival curves were drawn by employing the survival package and survminer package in R3.5.3.

### 2.9 Statistical analysis

To assess the prognosticative efficacy of the biomarkers, the receiver operating characteristic (ROC) curves were generated depending on the levels of gene expression and sample types. The log-rank test *p* values were calculated, and a *p* value of 0.05 or less was regarded as statistically significant. Software packages such as GraphPad Prism version 8.0 or SPSS version 20.0 were used to conduct the statistical analysis. Statistical significance was defined as a two-tailed *p* 0.05.

## 3 Results

### 3.1 Identification of DEGs during canine CRC formation

**Figure 1:**
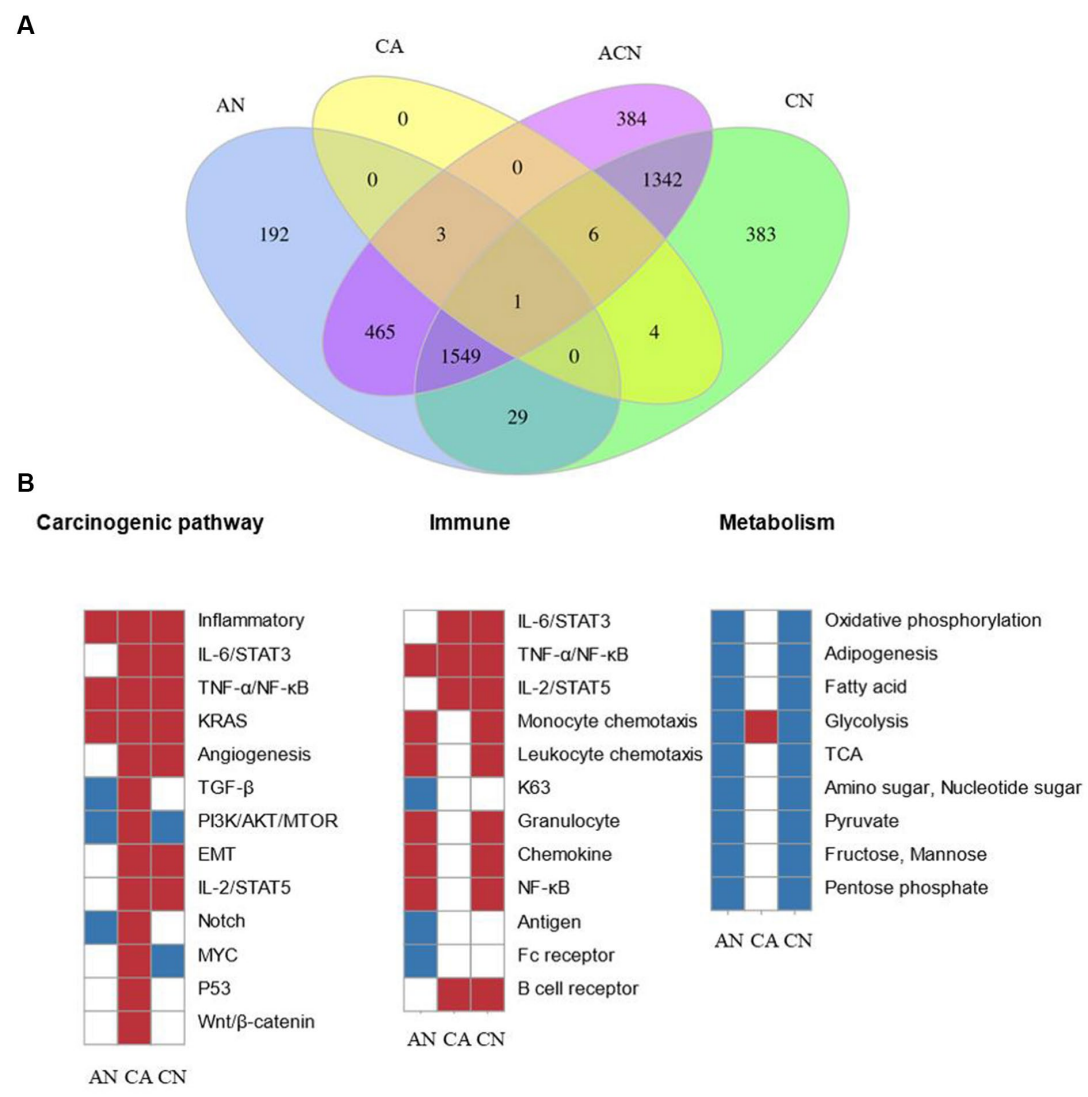
Comparing the transcriptome data at three types of tumor-identified DEGs and molecular characteristics of CRC formation. N, normal tissue; A, adenoma tissue; C, carcinoma tissue; AN, adenoma vs. normal colon; CA, carcinoma vs. adenoma; CN, carcinoma vs. normal colon; ACN, three tissue types. **(A)** Venn diagram showing the overlap of DEGs identified by AN (blue), CA (yellow), CN (green), and ACN (purple) comparisons. **(B)** GSEA heatmap showing significant changes in canonical pathway activity, metabolic, and immunological activities during CRC formation.

In total, 2,578 DEGs were identified by comparing adenoma with normal colon (AN), 3,701 DEGs were identified by comparing carcinoma with a normal colon (CN), whereas 14 DEGs were identified by comparing carcinoma with adenoma (CA), suggesting that adenomas were more similar to carcinoma than to the normal. 3,750 DEGs were found after comparisons of all three tissue types (ACN) by LRT (DEseq2) (Supplementary Tables S1–S4). Figure 1A displayed the overlapping DEGs between the normal colon, adenoma, and carcinoma tissues. PFKFB3 was the only gene that crossed across. Although comparisons can be performed between any two tissue types to identify DEGs that exhibit relative up- or down-regulation, it is challenging to identify changes in expression patterns across the adenoma–carcinoma sequence.

### 3.2 Early and late stages of canine CRC onset were related to three hallmarks

GSEA was used to compare the transcriptome patterns of each of the three different tissues. After reviewing previously published gene sets related to canonical pathway activity, and metabolic, and immunological processes, enriched gene sets (*p* < 0.05) were chosen to produce heatmaps, as shown in Figure 1B.

The stages of tumor formation were generally divided into the early stage or advanced stage. The early stage referred to the transformation of normal tissue into an adenoma, and the late stage referred to the transformation of adenoma tissue into carcinoma. Most typical pathways were activated during the transition from normal colon epithelial cells to adenomas to carcinoma, but only the TGF-β, PI3K/AKT/mTOR, and Notch pathways were downregulated during normal to adenoma transition and activated during the transition from adenoma to carcinoma. The inflammatory, TNF-α/NF-κB, as well as the KRAS pathways, were activated early during the transformation of normal to adenoma, and the IL-6/STAT3, angiogenesis, EMT, IL-2/STAT5, p53, and Wnt/β-catenin pathway were activated late during the transformation of adenoma to carcinoma.

Dysregulated metabolic activity was a feature of tumor formation. GSEA showed that the activity of most metabolism-related pathways decreased during tumor formation. Notably, glycolysis increased during the transformation of adenomas into cancerous ones.

During the adenoma to adenocarcinoma transition, genes affecting epithelial-mesenchymal transition (EMT) were significantly upregulated. Secondly, genes related to angiogenesis were significantly upregulated, which was consistent with the biological characteristics of the tumor. In addition, many classical pathways were up-regulated, including the response of genes regulated by NF-κB to TNF-α, the up-regulation of the STAT5 gene under the stimulation of IL2, and the up-regulation of genes after the activation of Notch, p53, and Wnt signaling pathway.

In the development and spread of malignancies, the effect of immune system was indispensable. Signaling pathways closely related to immunity, including IL-6/STAT3, TNF-α/NF-κB, and IL-2/STAT5, were activated during the transition.

Results from the GSEA showed that typical pathways, metabolism, and immune responses varied over time but were not always consistent. Different pathways underwent a variety of modifications at various transitions, some of which were engaged during the change from normal to adenoma and others involved in the transition from adenoma to carcinoma. We subsequently investigated the DEG expression patterns during tumor formation to shed further light on these findings.

### 3.3 Identification of dynamic expression patterns of adenoma to carcinoma processes

**Figure 2:**
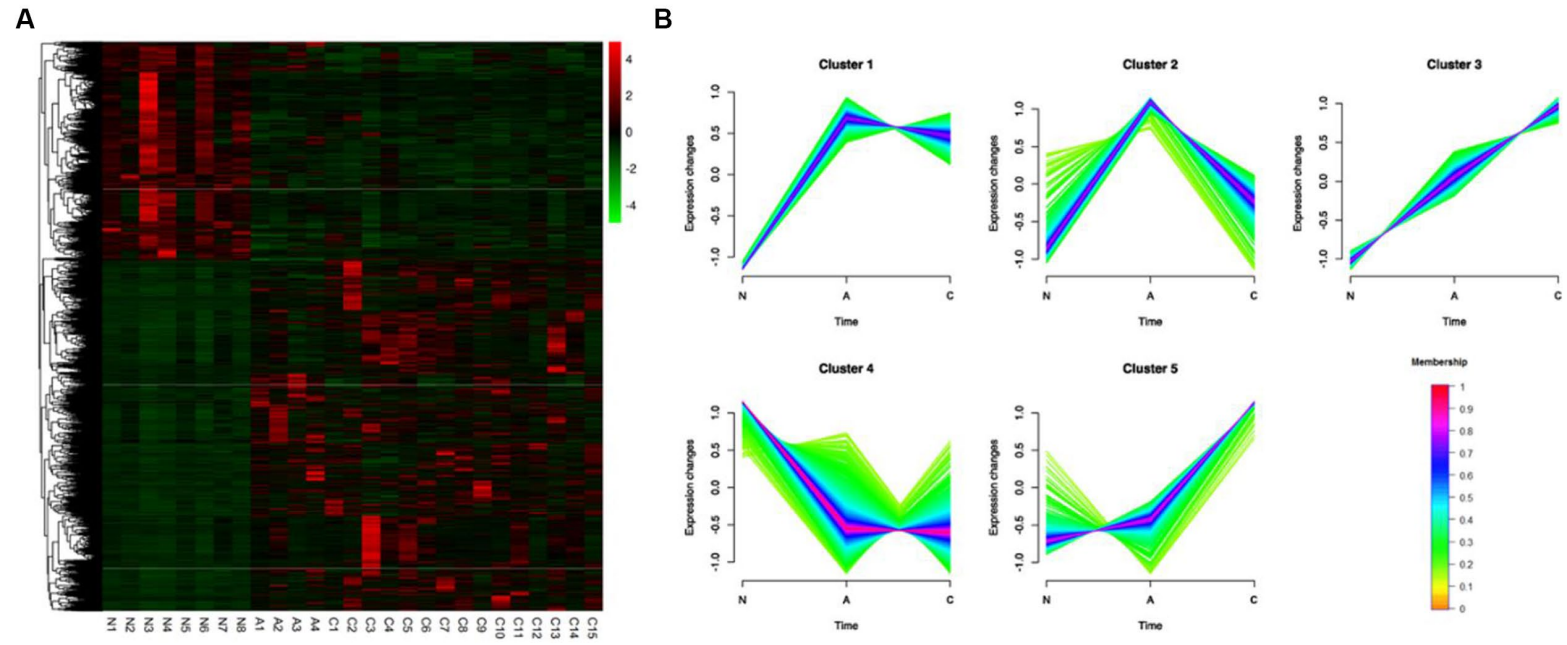
Identification of 3,750 DEGs dynamic expression profiles and chemical features from the ACN comparison. **(A)** 3,750 DEGs were heatmapped and clustered hierarchically. N, normal; A, adenoma; C, carcinoma. **(B)** The diagrams showed the patterns of dynamic changes in DEGs that were discovered using Mfuzz during the development of CRC.

Based on the comparison of the DEGs discovered by the three tissue types, unsupervised hierarchical clustering was carried out, and heat maps of 3,750 DEGs in 27 samples were created to comprehend the dynamic changes of DEGS in the three phases of CRC formation. As seen in Figure 2A, various tissue types were enriched for gene clusters with various degrees of expression. Using Mfuzz, the 3,750 DEGs were divided into five clusters in order to evaluate the dynamic expression patterns of DEGs during the beginning of CRC (Figure 2B) (Supplementary Table S5). The gene expression pattern of cluster 5 changed significantly in the later stage of tumor formation, while the upregulation of DEGs only occurred during the transformation of adenoma to malignancy. While DEGs were only up- or down-regulated during the transition from adenomas, the expression pattern of DEGs in clusters 1 and 4 changed considerably during the early stages of tumor formation. During the change from normal to cancerous tissue, DEGs in cluster 3 continued to steadily increase (Figure 2B).

### 3.4 Enrichment analysis associated with dynamic expression patterns

We hypothesized that different expression patterns of genes served diverse purposes throughout the development of CRCs. The five clusters’ GO and KEGG analysis revealed distinctive traits of the genes in each cluster.

GO analysis revealed that genes in cluster 1, which were upregulated during normal to adenoma transition, were associated with inflammation and immunity, including neutrophil chemotaxis, inflammatory response, chemokine-mediated signaling pathway, cellular response to interleukin-1, and the interleukin-17-mediated signaling pathway. Similar outcomes were found by KEGG analysis, which showed that genes were mainly involved in cytokine–cytokine receptor interaction, pathways in cancer, chemokine signaling pathway, inflammatory bowel disease, and MAPK signaling pathway (Supplementary Figure S1).

Genes in cluster 4, which were downregulated during normal to adenoma transition, were mainly associated with the metabolism (GO terms included hydrogen ion transmembrane transport, tricarboxylic acid cycle, lipid metabolic process, fatty acid beta-oxidation, aerobic respiration, mitochondrial ATP synthesis coupled proton transport, cholesterol biosynthetic process). KEGG analysis indicated that genes in cluster 4 were involved in pathways associated with the metabolic pathways, carbon metabolism, citrate cycle (TCA cycle), oxidative phosphorylation, chemical carcinogenesis – reactive oxygen species, among others (Supplementary Figure S4).

As shown in Figure 2D, genes in cluster 5, which were upregulated during the adenoma to carcinoma transition, were mainly involved in canonical pathways associated with cancer, including the Ras, PI3K-Akt, Proteoglycans in cancer, MAPK, and Hippo signaling pathways, among others (Supplementary Figure S5).

GO analysis revealed that genes in cluster 3, which were monotonously upregulated during the normal-adenoma–carcinoma sequence, were associated with immunity and cancer, including inflammatory response, positive regulation of cytokine production, signal transduction, chemotaxis cytokine-mediated signaling pathway, positively regulated inflammatory responses, IL-6 production, and cell–cell signaling. Similar conclusions were drawn from KEGG analysis, which revealed that genes were primarily engaged in the pathways for NF-κB signaling, IL-17 signaling, TNF signaling, pathways in cancer, and JAK-STAT signaling. Supplementary Figure S3 displayed the findings of GO and KEGG analysis of the other clusters (Supplementary Figure S3).

### 3.5 Construction of the co-expression network and identification of hub modules related to pathological phenotype using weighted gene co-expression network analysis

**Figure 3:**
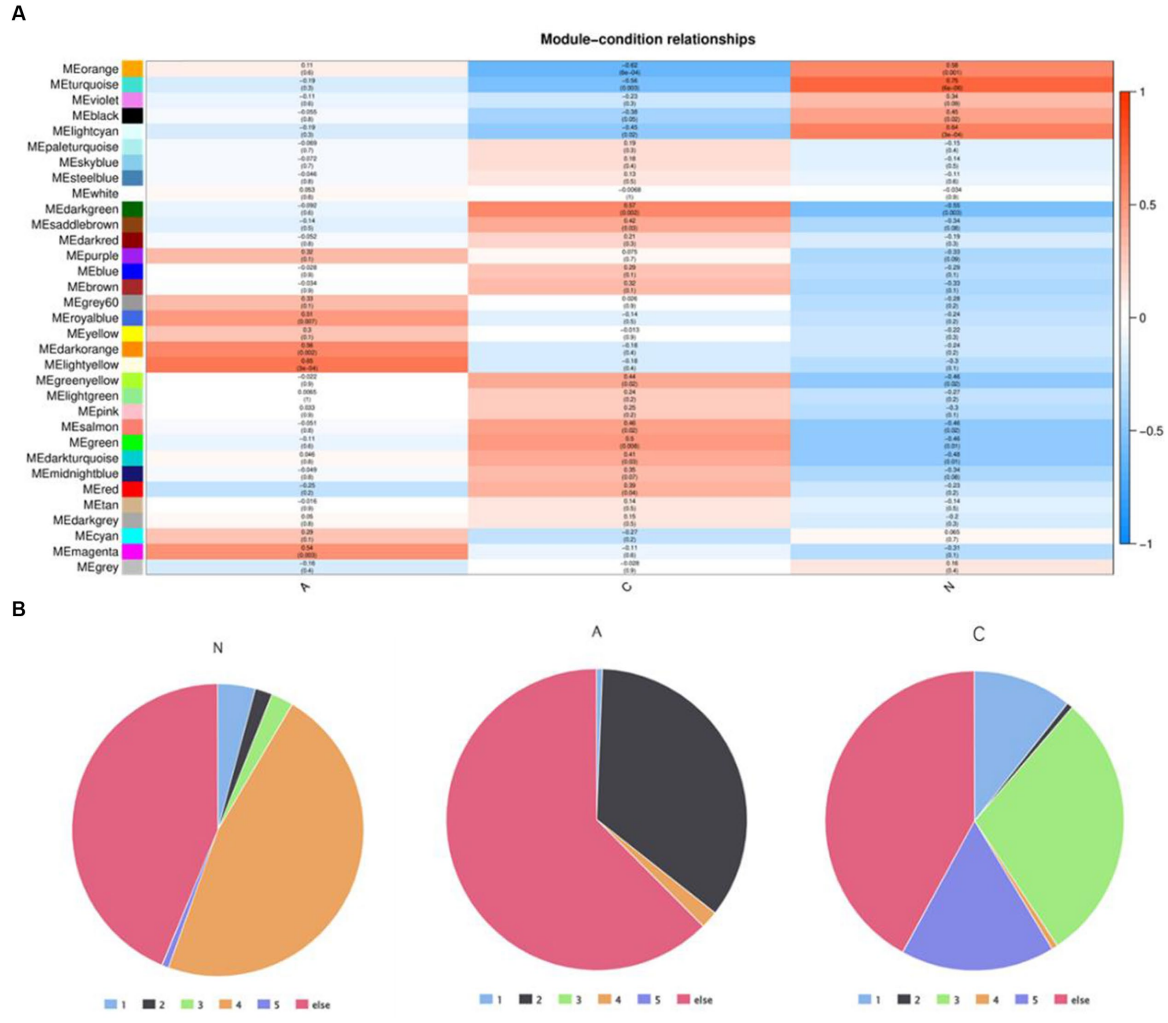
**(A)** Weighted gene co-expression network analysis identified the module genes closely related to tumor pathologic phenotypes and **(B)** distribution of DEGS in core module genes in five dynamic expression modes.

WGCNA was applied to 12,866 genes with an expression of at least 0.1 FPKM in order to investigate the association between DEGs and the pathophysiology of tissues. This process produced 33 modules of highly co-expressed gene modules. Three of the modules’ DEGs had a strong correlation with tissue phenotype. DEGs were closely linked to malignancy in the darkgreen module (cor = 0.57, *p* = 0.002) (Supplementary Table S6), benign in the lightyellow module (cor = 0.65, *p* = 3e-04), and normal tissues in the turquoise module (cor = 0.75, *p* = 6e-06) (Figure 3A). DEG expression patterns were dynamic in these three modules. Clusters 4 (1,261/2684) in the turquoise module were enriched in DEGs that were linked to adenoma tissues. The genes in the darkgreen module that were linked to malignant tissues were more prevalent in clusters 1 (15/150), 3 (44/150), and 5 (25/150).

The differentially expressed genes in cluster 4 may play a key role in the progression of normal tissue to adenoma. The proportion of differentially expressed genes in cluster 4 is the largest in the normal module, but the proportion in both benign and malignant modules is very small. Meanwhile, the proportion of genes in cluster 2 is very small in both normal and malignant modules, but the proportion in the benign module is very high. This suggests that genes in clusters 2 and 4 could be engaged in the essential early events of tumor formation but may not be the key clusters affecting tumor progression.

The genes in cluster 3 and cluster 5 had the highest proportion in the malignant module and a low proportion in both the normal and benign modules, suggesting that these genes could be crucial for the eventual deterioration of the organization.

Through the analysis of this gene enrichment, this finding implies that in the development of colorectal cancer in dogs, the first is the dysregulation of metabolic activity (metabolic reprogramming), combined with the loss of cell proliferation and tight connections, and the development of normal tissues into adenomas. In further development, the Wnt signaling pathway, IL-6/STAT3 signaling pathway, changes in tumor immune microenvironment, and epithelial-mesenchymal transformation play an important role in the malignant transformation process of tissues.

### 3.6 Dynamic expression patterns associated with pathological phenotype in the adenoma–carcinoma sequence

**Figure 4:**
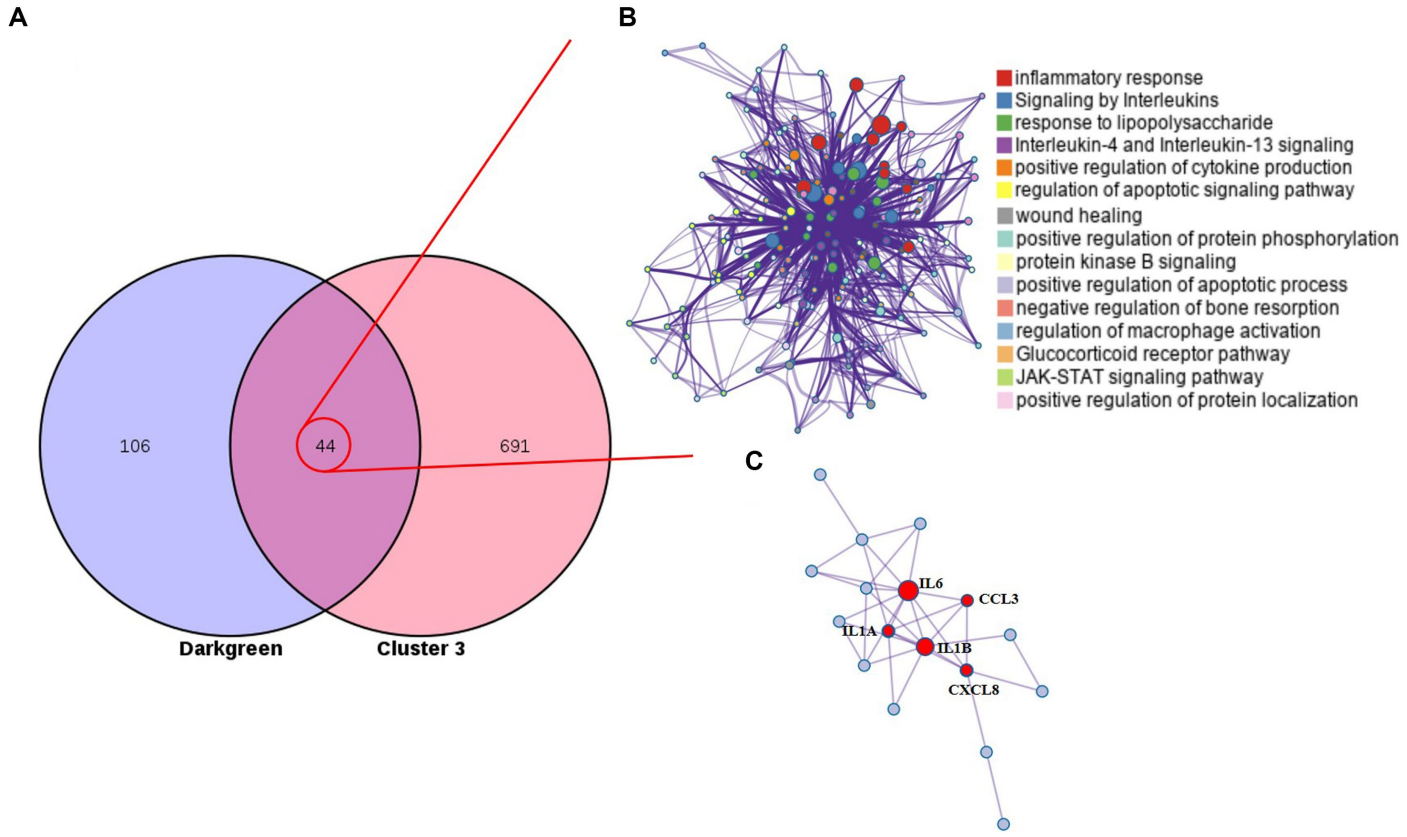
Cluster 3 genes in the core module. **(A)** The Venn diagram found 44 overlapping genes. **(B)** PPI network and enrichment analysis of 44 genes. **(C)** The core genes identified by MCODE.

The monotonous changes following the formation of CRC indicated that these genes may be suitable as specific tracking biomarkers or driving factors. DEGs in cluster 3 were selected for further analysis as markers of CRC formation. The Venn diagram identified 44 genes that were consistently up-regulated in the modules significantly related to the malignancy of tumors, and the proteins represented by these genes had complex interactions (Figures 4A,B). Enrichment analysis found that these genes were mainly related to inflammation and immunity, suggesting that during the occurrence and development of colorectal cancer in dogs, changes in the tumor immune microenvironment may play the most important role. Through the core modules identified by MCODE, we found two main communities, one of which is the immune function community with IL6 as the core, and the other is the community with the S100 protein family as the core (Figure 4C). The biological functions of these core genes have been extensively studied in human tumor diseases, but need to be further explored in human and canine colorectal tumors. Cibersort was used to analyze the infiltration of immune cells in the three types of tissues. It was found that the infiltration of monocytes and neutrophils in the tumor tissues was increased, and the decrease of activated NK cells, the decrease of CD8^+^T cells, and the increase of regulatory T cells all suggested the phenomenon of immunosuppression in canine colorectal tumors (Supplementary Figure S6).

### 3.7 Dog–human comparison strategy reveal core genes

**Figure 5:**
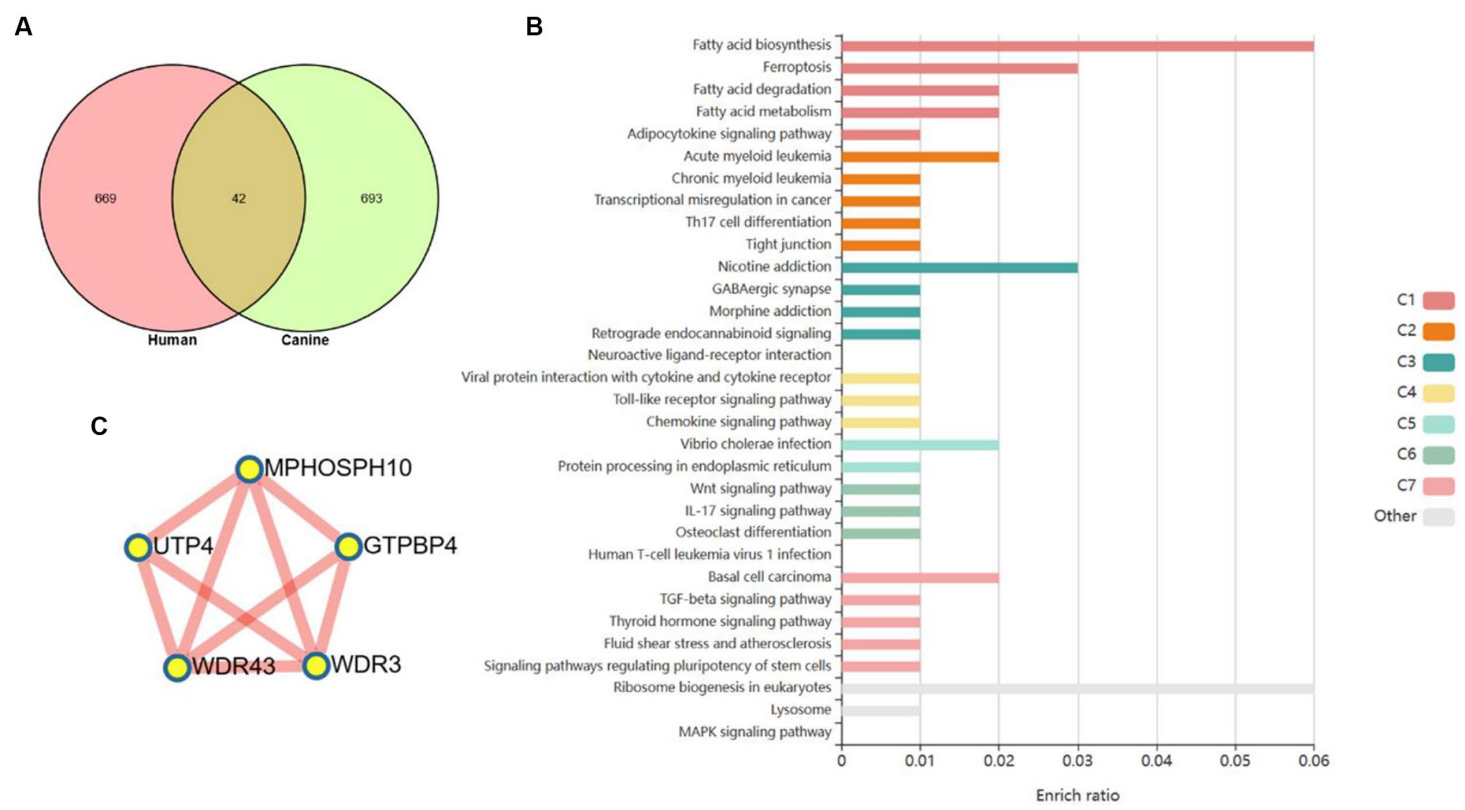
Core genes were screened based on human–dog comparative strategy. **(A)** Venn diagram to get the core genes. **(B)** Enrichment analysis reveals a variety of pathways and biological processes. **(C)** PPI network was used to screen out more core differential genes.

Based on a new strategy for comparing humans and dogs across species, it could help clarify a central goal of cancer research, namely cancer driver–passenger distinctions. We compared to cluster 3 with clusters with the same expression pattern in the dynamic pattern of human colorectal adenoma-cancer and obtained 42 differential genes (Figure 5A). These differential genes are based on cross-species comparisons and are more likely to drive the development of cancer. The enrichment analysis of 42 differential genes revealed a variety of biological processes closely related to cancer. At the metabolic level, it involves the biosynthesis, decomposition and metabolism of fatty acids. It is involved in TH17 differentiation, IL17 signaling pathway and cytokine signaling pathway at the immune level. In addition, several classical cancer-related signaling pathways are involved, including the toll-like receptor, chemokine, Wnt, TGF-β, and MAPK signaling pathways (Figure 5B). A PPI network of 42 differential genes was constructed according to the STRING database, and 5 core genes that may play a key role were obtained by further analysis of MCODE (Figure 5C).

### 3.8 Diagnostic and survival analyses revealed the efficacy of five potential markers

**Figure 6:**
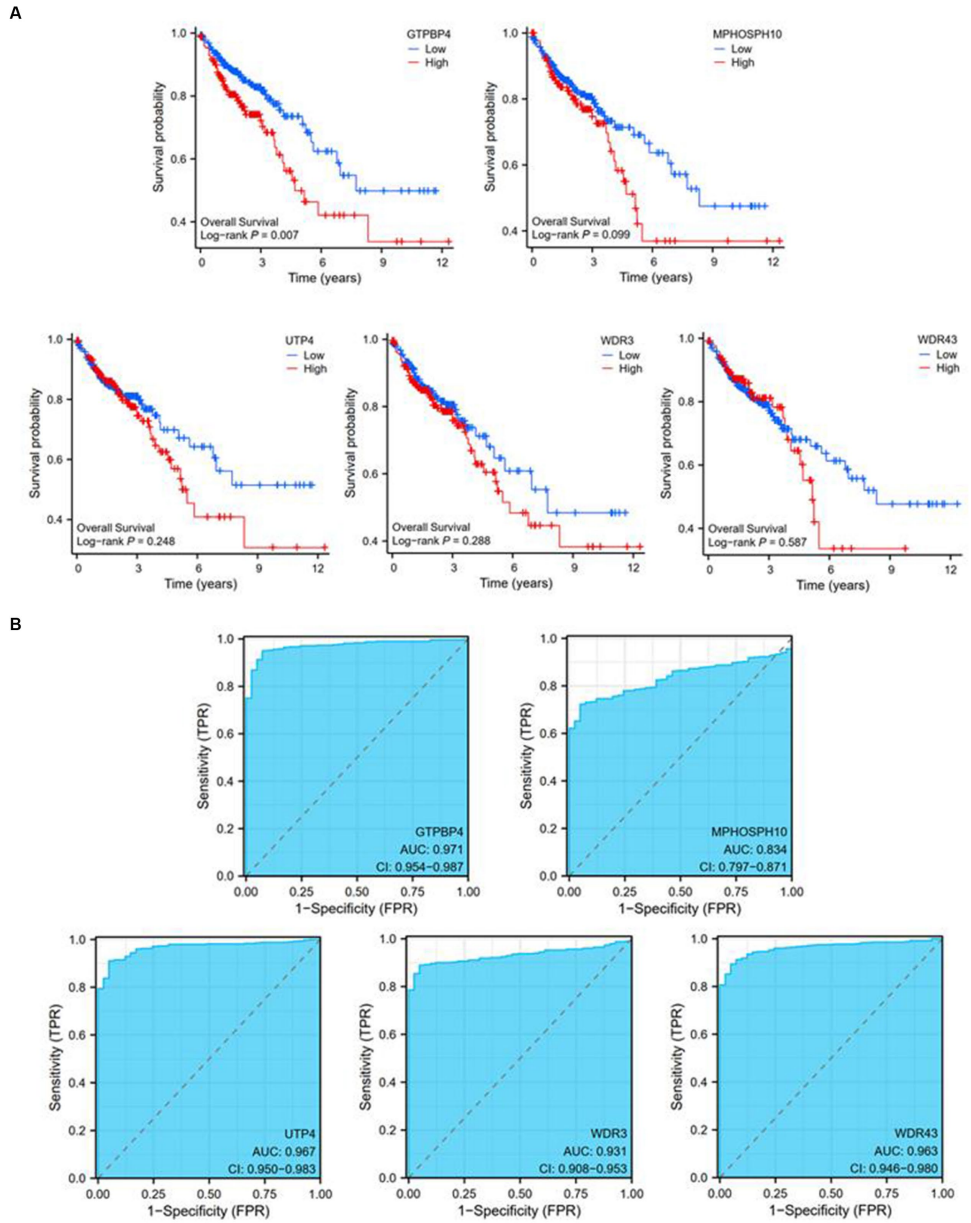
Diagnostic and prognostic ability tests of five genes. **(A)** Survival curve of GTPBP4, MPHOSPH10, UTP4, WDR3, and WDR43. **(B)** ROC curve of GTPBP4, MPHOSPH10, UTP4, WDR3, and WDR43.

Utilizing expression data and survival data from the TCGA database, we assessed the diagnostic and prognostic utility of the five DEGs. The likelihood of OS was calculated using a Kaplan–Meier analysis, and groups with various levels of gene expression were compared using the log-rank test. High expressions of GTPBP4 were correlated with poor survival (Figure 6A). ROC curve analysis showed that all of them could be used as accurate biomarkers to assist in the identification of colorectal tumors. And GTPBP4 had the highest AUC score and the best diagnosis effect (Figure 6B).

## 4 Discussion

This evolutionary process of adenoma–carcinoma is widely accepted in human colorectal cancer studies (22). Based on the numerous molecular homologies and clinical characteristics of colorectal tumors in humans and dogs, canine colorectal tumors are likely to follow this evolutionary process (23). The dynamic expression pattern of human colorectal cancer has been extensively studied, but not reported in dogs.

We found that in adenoma and carcinoma, the expression of the number of gene activation is generally more than the number of gene expression suppression and four kinds of DEG eventually involve PFKFB3, which aroused our interest. Targeting glycolytic fluxes by metabolizing PFKFB3 for the treatment of glucose-dependent cancers. PFKFB3 increased IL-1β and TNF-α in intestinal epithelial cells to promote colitis-related colorectal cancer tumorigenesis. Interleukin-6 stimulated aerobic glycolysis by regulating PFKFB3 at the early stages of colorectal cancer (24). Mir-488 alleviated chemical resistance and glycolysis in colorectal cancer by targeting PFKFB3 (25). There are no studies exploring the biological role of PFKFB3 in canine colorectal tumors, nor even a single paper on canine topics, which is a good direction for future exploration.

The genes in cluster 5 of the five dynamic expression patterns were only altered when an adenoma turned into a carcinoma, and they were particularly enriched in the usual cancer pathway. Any gene that exhibited cluster 5’s dynamic expression pattern during the development of a tumor may be crucial to the malignant transformation process. This methodology might make it easier to find new targets for cancerous transformation. GO and KEGG’s analysis found that immune dysregulation played a key role in the development of tumors. Many immune pathways were primarily related to innate immunity. In the past decades, tumor immunity research has ignored the contribution of innate versus adaptive immunity. Additionally, mounting data indicates that innate immunity may be crucial to the carcinogenic effects of bowel cancer (26). Therefore, the application of adaptive and innate immune systems to fight with CRC cells could overcome the specificity problem, which was a significant concern in chemotherapy and radiotherapy (27).

Among the five dynamic expression patterns, the genes in cluster 5 were only changed during the transition from adenoma to cancer and were enriched in the typical cancer pathway. Any gene that follows the dynamic expression pattern of cluster 5 during tumor formation may play a key role in malignant transformation. This model may help facilitate the discovery of new targets for malignant transformation. GO and KEGG’s analysis found that immune dysregulation played a key role in the development of tumors. Many immune pathways were primarily related to innate immunity. In the past decades, tumor immunity research has ignored the contribution of innate versus adaptive immunity. Increasing evidence also suggests that innate immunity may play an important role in the carcinogenic effects of bowel cancer (26). Therefore, the application of adaptive and innate immune systems to fight with CRC cells could overcome the specificity problem, which was a significant concern in chemotherapy and radiotherapy (27).

Combined with WGCNA analysis, we found that immune-related genes may play a key role in the malignant progression of colorectal tumors. As a weighted target, IL-6 has been extensively studied in human colorectal cancer. We were also surprised to find that the S100A protein family played an important role in the malignant development of canine colorectal tumors.

Strategies based on cross-species comparisons are more likely to identify cancer drivers, and we followed the new strategy of human–dog comparisons. In fact, a similar gene expression pattern exists in human colorectal cancer, and the consistently up-regulated gene community is more likely to play an important role in cancer. Through comparative screening, we found 42 common differential genes, and enrichment analysis found that these genes have a wide range of biological roles related to classical cancer pathways, metabolism and immunity are covered.

Five core genes were identified by further analysis of the 42 genes, which were GTPBP4, MPHOSPH10, UTP4, WDR3, and WDR43.

A critical regulator of cell cycle progression and MAPK activation is guanosine triphosphate binding protein 4 (GTPBP4), a member of the GTPBP family that is highly conserved throughout eukaryotes from yeast to humans (28). It was demonstrated that GTPBP4 promoted hepatocellular carcinoma (HCC) growth and metastasis both *in vivo* and *in vitro* and promoted aerobic glycolysis by inducing dimeric pyruvate kinase M2 (PKM2) formation (29). A higher level of GTPBP4 was detected in CRC metastatic tissues, and GTPBP4 has been proven to promote CRC metastasis by disrupting RhoA activity (30). GTPBP4 has shown superior diagnostic and survival effects through human colorectal cancer survival analysis and diagnostic tests.

M-phase phosphoprotein 10 (MPHOSPH10) belonged to categories of cellular physiologic response and signal transduction (31), however, its role in tumor progress has still been little studied. It was found that hypoxia markedly down-regulated cell survival-related genes such as, MPHOSPH10, IMP-3, ITGA2, SDCBP, and IGBP3 in SK-N-MC cells (32).

One of the subcomplexes in the small subunit (SSU) processome, the U three proteins (Utps), was essential for the formation of ribosomal chromatin (r-chromatin) and for the efficient production of rDNA (33). Seven proteins made up the t-Utp subcomplex: Utp4, Utp5, Utp8, Utp9, Utp10, Utp15, and Utp17 (34). Utp4 was a ribonucleoprotein complex required for ribosomal RNA processing and small subunit assembly (35).

The tryptophan-aspartate repeat (WDR) domain was engaged in a large number of cellular processes, such as the ubiquitin-proteasome system (UPS), the G protein-coupled receptor signaling pathway, DNA damage perception and repair, epigenetic chromatin regulation, and the immune system (36). WD repeat domain 3 (WDR3), also known as DIP2 or UTP1, a member of the WD-repeat family, was engaged in a number of cellular processes, including signal transmission, apoptosis, gene regulation, and cell cycle progression (37, 38). By interacting with GATA4, WDR3 activated the Hippo signaling pathway, demonstrating that it was crucial in promoting the advancement of pancreatic cancer (39). Additionally, prostate cancer (PCa) tissues were found to have a substantially higher WDR3 level, and WDR3 overexpression increased markers of stem cell-like characteristics (40). These studies above showed that WDR3 may assist some malignant cancers to grow and proliferate, nevertheless, the biological function of WDR3 in CRC and its associated mechanism were still unknown.

Tryptophan-aspartate repeat domain 43 (WDR43), the ortholog of yeast Utp5, could interact with the Pol II machinery in embryonic stem cells (ESCs) (41, 42). By examining the data from the Gene Expression Omnibus (GEO) dataset and The Cancer Genome Atlas (TCGA) database, WDR43 was identified as a potentially significant oncogenic factor in the pathogenesis of CRC and a marker for predicting the efficacy of chemotherapy (43), which was consistent with our results. Additionally, cancer cells with higher WDR43 expression were more resistant to chemotherapy-mediated cell death and therefore the overexpression of WDR43 was related to the poor prognosis of CRC patients. *In vitro* studies have revealed that WDR43 knockdown increased apoptosis, inhibited CRC cell proliferation, migration, and invasion, and slowed carcinogenesis in animal models (44).

In pan-cancer analysis, these genes were significantly expressed differently in multiple cancers (Supplementary Figure S7), which reinforced our belief in the significance of this finding. The important role of GTPBP4, MPHOSPH10, UTP4, WDR3, and WDR43 in human tumors has been discovered, but more in-depth research on the mechanism is still lacking. However, in the study of canine CRC, the molecular functions and clinical diagnostic predictiveness of these markers have not been proven, which deserved further exploration.

## 5 Conclusion

Our study was the first to explore this dynamic expression pattern of normal-adenoma–carcinoma in canine CRC, explaining the molecular characteristics of colorectal tumor development in dogs. In conclusion, GTPBP4, MPHOSPH10, UTP4, WDR3, and WDR43 could serve as valuable biomarkers of cross-species values and provide new selections for the future diagnosis and treatment for both human and canine CRC.

## Data availability statement

The original contributions presented in the study are included in the article/Supplementary material, further inquiries can be directed to the corresponding authors.

## Ethics statement

Ethical approval was not required for the study involving animals in accordance with the local legislation and institutional requirements because this study only analyzed publicly available sequencing data and did not involve animal testing.

## Author contributions

ZL designed experiments and analyzed the data. JZ drafted the manuscript. QC and XZ conducted the experiments. JL reviewed the manuscript. JL and DL supervised the project. ZL and DZ conceived the project. All authors contributed to the article and approved the submitted version.

## Footnotes

^1^ https://www.gsea-msigdb.org/gsea/index.jsp

^2^ https://portal.gdc.cancer.gov/
