# Supplementary material for: Transcriptome analysis of the adenoma–carcinoma sequences identifies novel biomarkers associated with development of canine colorectal cancer
Source: Front Vet Sci. 2023 Nov 29;10:1192525. doi: 10.3389/fvets.2023.1192525 (PMC10720982; doi:10.3389/fvets.2023.1192525)
Supplement: Supplementary file 1 [file Data_Sheet_1.ZIP › Supplementary_Material.docx]

Supplementary Material

Network pharmacology and Transcriptomics analysis unveil the anti-colon cancer mechanisms of Rhein-Ligustrazine Derivative

Zixiang Lin1†, Qi Chen1†, Jiatong Zhang1, Xiaohu Zhang1, Di Zhang1*, Jiahao Lin1,2*, Degui Lin1*

1 Department of Veterinary Clinical Sciences, College of Veterinary Medicine, China Agricultural University, Beijing 100193, China.

2 Department of Center of Research and Innovation of Traditional Chinese Veterinary Medicine, China Agri-cultural University, Beijing 100193, China.

* Correspondence:

De-gui Lin, ldgcau@sina.com; Jia-hao Lin, jiahaolin@cau.edu.cn.; Di Zhang, [dzhangdvm@cau.edu.cn](mailto:dzhangdvm@cau.edu.cn)

**Supplementary Figures**

**
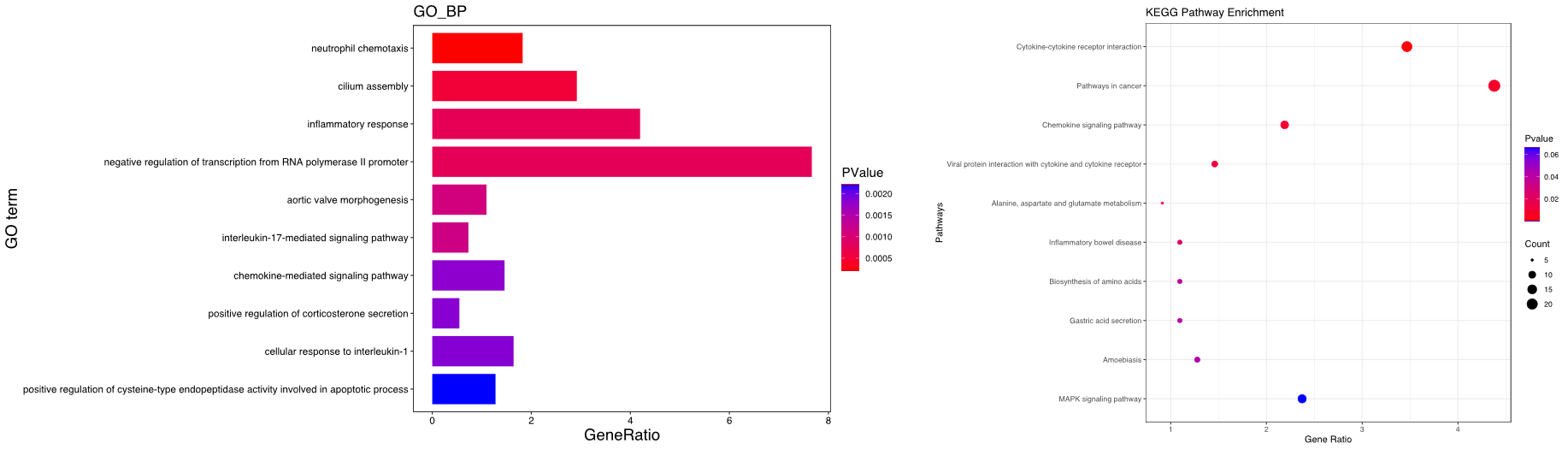
**

**Supplementary Figure 1. GO and KEGG enrichment analysis of cluster 1 genes**


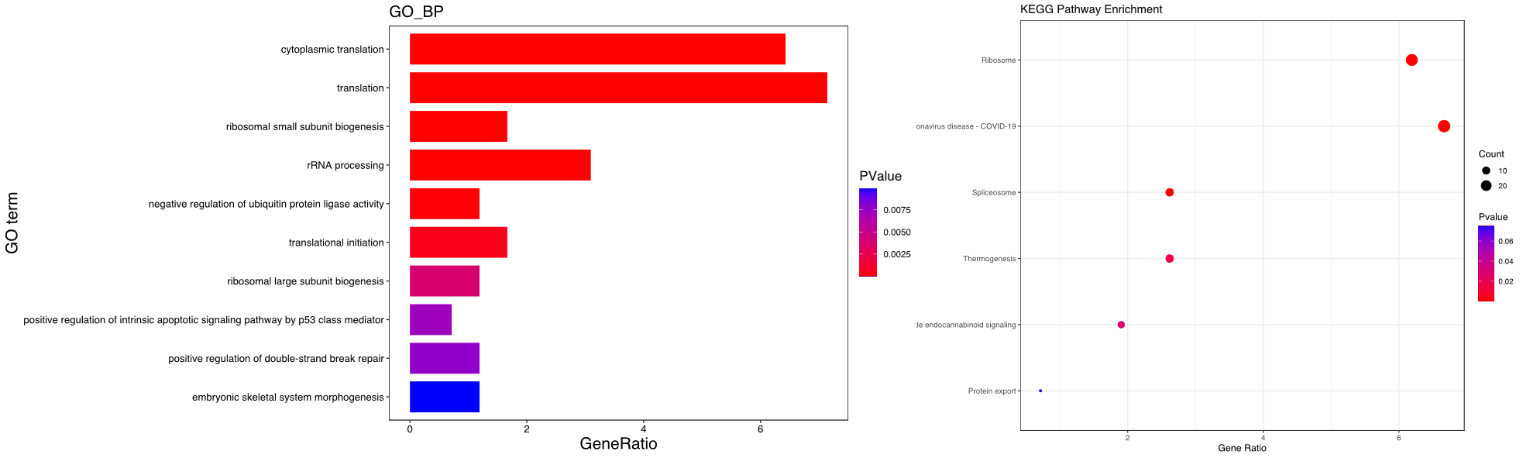


**Supplementary Figure 2.** GO and KEGG enrichment analysis of cluster 2 genes


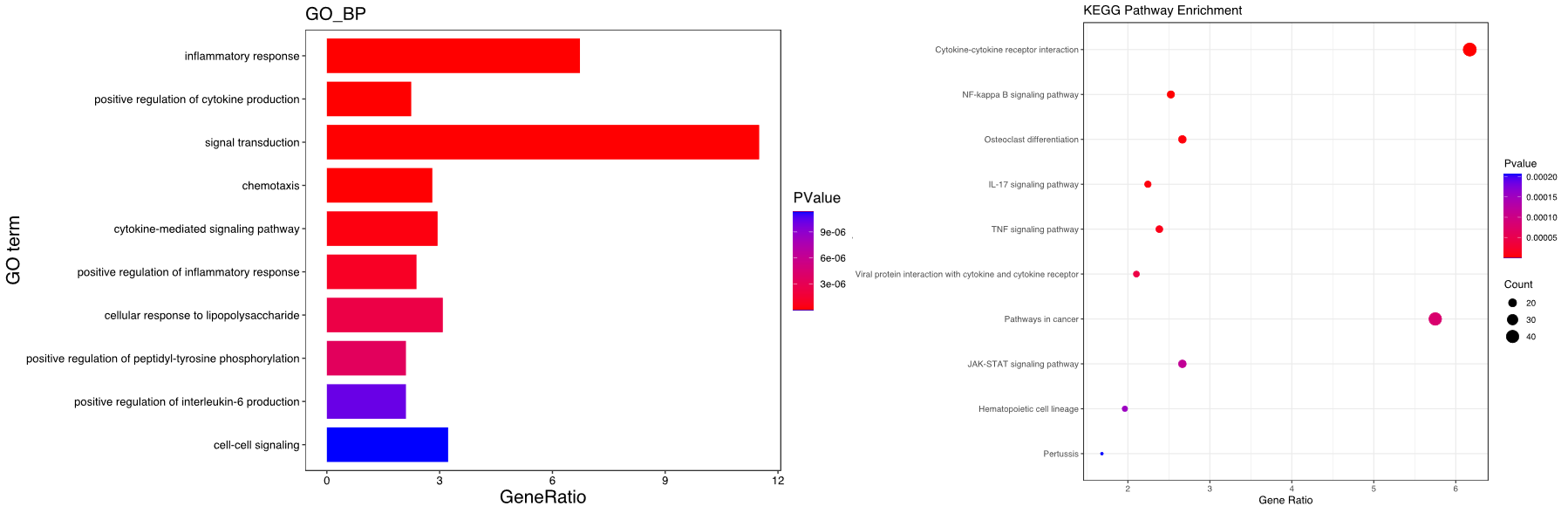


**Supplementary Figure 3.** GO and KEGG enrichment analysis of cluster 3 genes


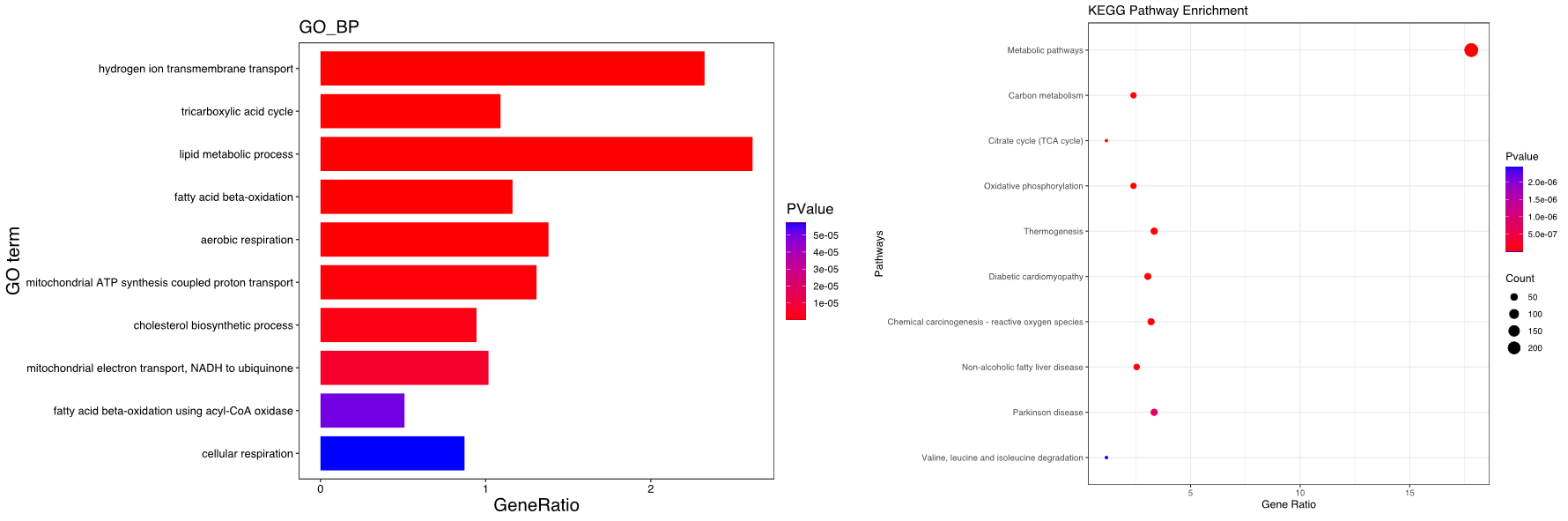


**Supplementary Figure 4.** GO and KEGG enrichment analysis of cluster 4 genes


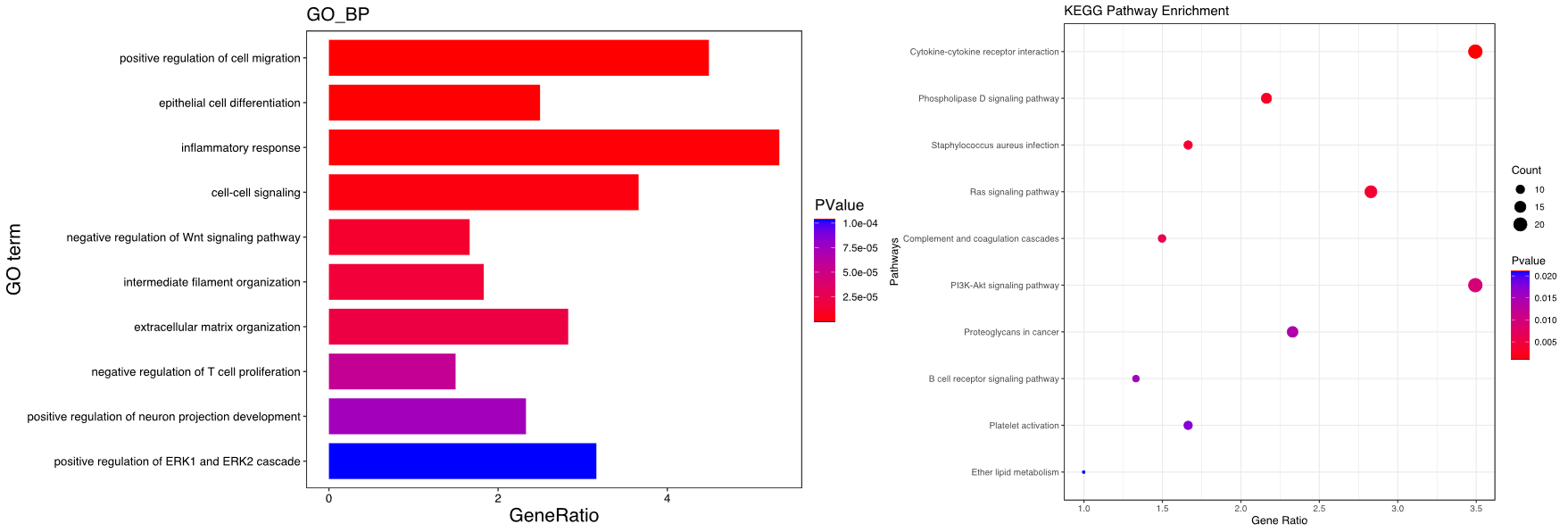


**Supplementary Figure 5.** GO and KEGG enrichment analysis of cluster 5 genes


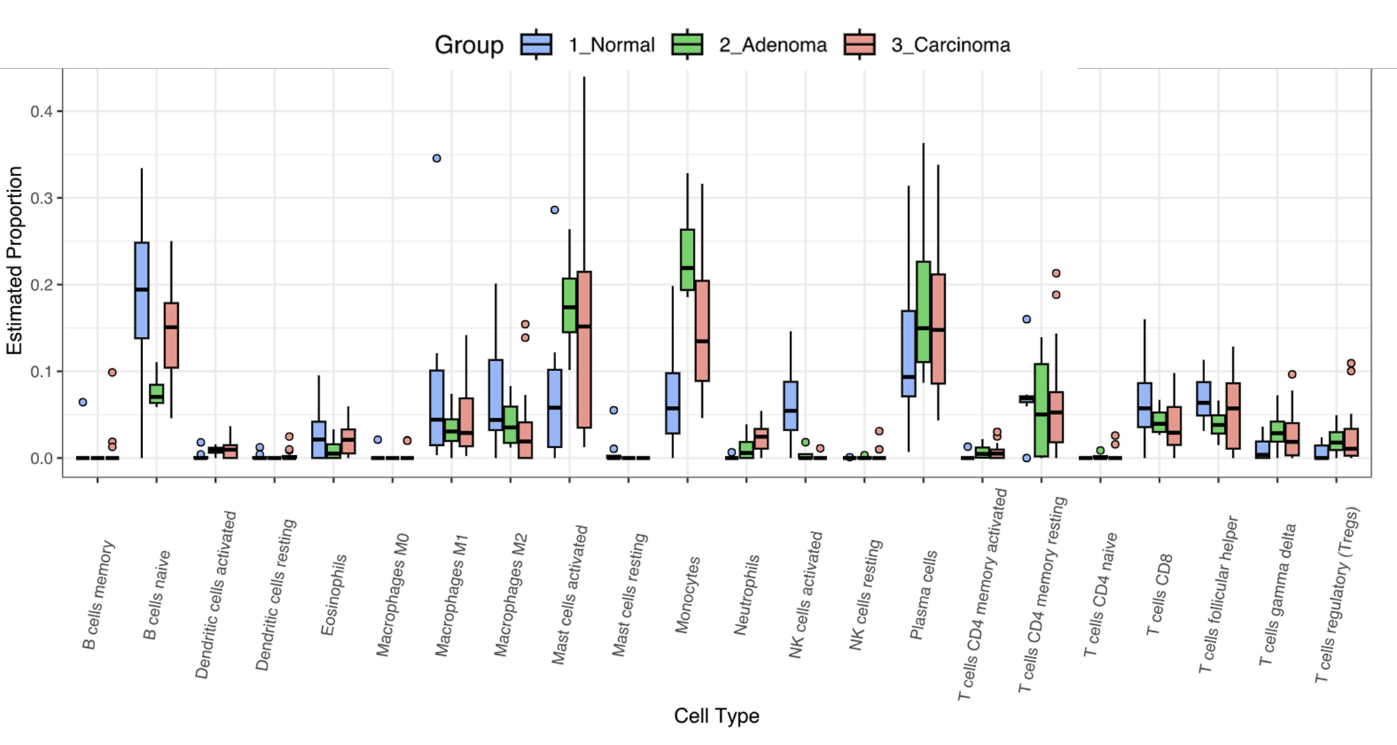


**Supplementary Figure 6.** Infiltrating state of immune cells in normal colo-adenoma-carcinoma tissue in dogs


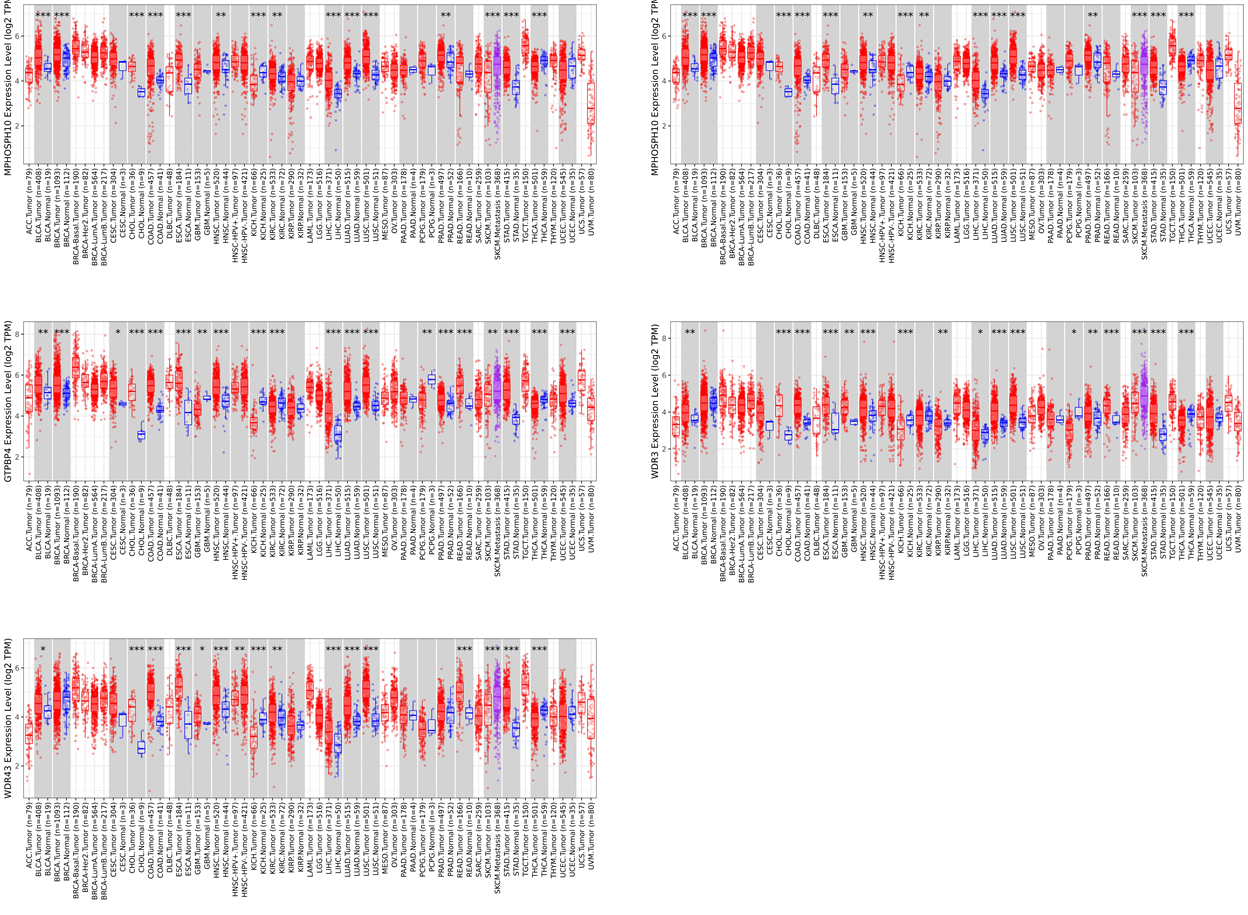


**Supplementary Figure 7.** Expression of five genes in pan-carcinoma analysis
